# Transcriptomic Analysis of the Effect of Metformin against Cisplatin-Induced Ototoxicity: A Potential Mechanism of Metformin-Mediated Inhibition of Thioredoxin-Interacting Protein (Txnip) Gene Expression

**DOI:** 10.3390/cimb45010021

**Published:** 2022-12-31

**Authors:** Sehee Lee, Sun Choi, Seok Hyun Park, Gi Jung Im, Jiwon Chang

**Affiliations:** 1 Department of Otorhinolaryngology-Head & Neck Surgery, Hallym University College of Medicine, Kangnam Sacred Heart Hospital, Seoul 07441, Republic of Korea; 2 Department of Otorhinolaryngology-Head & Neck Surgery, Korea University College of Medicine, Seoul 02841, Republic of Korea

**Keywords:** cisplatin, ototoxicity, metformin, prevention, gene expression, Txnip pathway

## Abstract

Ototoxicity is the drug-induced damage of the inner ear, causing bilateral irreversible sensorineural hearing loss. Cisplatin is a widely used chemotherapeutic agent which causes ototoxicity as its side effect. Pretreatment with metformin prior to the application of cisplatin significantly decreased the late apoptosis and attenuated the cisplatin-induced increase in ROS. To understand the molecular mechanisms that are involved in the preventive effect of metformin, we evaluated the change of gene expression induced by cisplatin at several different time points (0 h, 6 h, 15 h, 24 h and 48 h) and the alteration of gene expression according to pretreatment with metformin in HEI-OC1 cells through microarray analysis. Cisplatin exposure induced a total of 89 DEGs (differentially expressed genes) after 6 h, with a total of 433 DEGs after 15 h, a total of 941 DEGs after 24 h, and a total of 2764 DEGs after 48 h. When cells were pretreated with metformin for 24 h, we identified a total of 105 DEGs after 6 h of cisplatin exposure, a total of 257 DEGs after 15 h, a total of 1450 DEGs after 24 h, and a total of 1463 DEGs after 48 h. The analysis was performed based on the gene expression, network analyses, and qRT-PCR, and we identified several genes (CSF2, FOS, JUN, TNFα, NFκB, Txnip, ASK1, TXN2, ATF3, TP53, IL6, and IGF1) as metformin-related preventive biomarkers in cisplatin ototoxicity.

## 1. Introduction

Sensorineural hearing loss is irreversible and damaged inner ear hair cells do not regenerate after the loss. In avian, cochlea and vestibular hair cell is reported to regenerate after the exposure to noise or ototoxic drugs. However, the inner ear regeneration is scare in mammals, and it is not until recently that neonatal mouse cochlea harbor cells that are capable of regeneration within a week after birth. Therefore, numerous research regarding inner ear hair cell loss due to age, noise exposure, ototoxicity and so on, has focused on the prevention or the protection of these sensory organ.

Ototoxicity is a drug-induced damage of inner ear causing bilateral progressive irreversible sensorineural hearing loss, tinnitus and imbalance. Cisplatin is widely used chemotherapeutic agents but one of its side effects is ototoxicity with the average incidence of 62–100% [1,2]. Hearing loss has severe impacts on the quality of life. It prevents communication and participation in social networks. It has adverse effects on individual’s cognition, mental health and reported to be related to dementia. Additionally, when it occurs in very young children, it delays speech development and seriously affects cognitive and psychosocial development. Thus, it is important to treat or prevent the hearing loss caused by use of cisplatin. In the research regarding the cisplatin ototoxicity, it is important to identify the protective agents or the mechanism that maintain the drugs’ role as a treatment agent but to reduce their side effects. 

Several antioxidants have provided efficacy in reducing cisplatin-induced hearing loss in animal models [3,4]. Drugs containing thiol group such as N-acetyl cysteine, sodium thiosulfate, D-methionine, and lipoic acid have a high affinity for cisplatin and reported to have some protective effects [3,4]. A calcium channel blocking agent, flunarizine, reduced cell death by activation of antioxidant protective mechanisms in the cochlea Nrf2 (Nuclear factor erythroid-2-related factor 2) and heme oxygenase-1 [5], and inhibited inflammatory pathways by reducing the activity of NF-kB (nuclear factor kB) [5,6]. The adenosine A1 receptor agonist R-PIA was reported to have protect effect against cisplatin ototoxicity in the rat by exerting an anti-inflammatory effect by preventing ROS (reactive oxygen species) from being generated by the NOX3 (NADPH oxidase 3) enzyme and by downregulation of the STAT-1 (signal tranducer and activator of transcription 1) inflammatory pathway [7]. The cannabinoid 2 (CB2) receptor agonist protected against cisplatin ototoxicity by inhibition of STAT1, thereby preventing cell death in the cochlea [8]. Intratympanic application of siRNAs against TRPV1, NOX3, and STAT1 provided protection against cisplatin ototoxicity in rat model by decreasing ROS generation and preventing inflammation in the cochlea [3]. 

There are also several promising agents for protection against cisplatin-induced hearing loss. Clinical studies on sodium thiosulfate [9,10], intratympanic N-acetylcysteine injection, intratympanic dexamethasone injection [11,12,13], amifostine [14,15,16] and vitamin E [17] are on the way, but currently, there are no FDA-approved treatments available. It would be promising if it is possible to identify the inner ear protective effects of pre-existing medications or agents which do not interfere with cisplatin’s tumor-killing efficacy. 

Metformin is a conventional therapeutic medication for type II diabetes mellitus to control the glucose level. Other than the drug’s role as an anti-diabetic drug, previous studies reported that metformin has shown to prevent oxidative stress-induced cell death [18], has been considered as a potent anticancer drug and mostly [19], and has gained significant attention as an anti-aging drug [20]. Key signaling pathways involved in senescence focus on AMP-activated protein kinase (AMPK) and mTOR (mammalian target of rapamycin) [21]. AMPK plays a crucial part in tissue energy and in immune response, and the activation of the AMPK signaling pathway inhibits various relevant immune signaling pathways such as NF-κB, JAK/STAT, C/EBPβ, HIF-1α and CHOP pathways. Another important pathway involves mTOR and mTOR is a highly conserved serine/threonine protein kinase, which controls the growth of cells and induced by growth factors and nutrients. Metformin is known to exert direct immunomodulatory effect on immune cells by AMPK induction and mTOR inhibition.

In our previous studies with metformin, pretreatment with metformin prior to the application of cisplatin significantly decreased the frequency of late apoptosis in HEI-OCI cells [22]. Metformin attenuated the cisplatin-induced increase in ROS, inhibited the activation of caspase-3 and levels of poly-ADP-ribose polymerase (PARP) and prevented the cisplatin-induced elevation in intracellular calcium concentrations. However, AMPK and mTOR related pathways which are the main mechanism of metformin were not identified in our previous study. 

In this study, we evaluated the change of gene expression induced by cisplatin at several different time points, and the alteration of gene expression according to pretreatment with metformin in HEI-OC1 cells through microarray analysis. We also identified potential molecular modulator genes and pathways to determine the role of metformin in the cisplatin ototoxicity. 

## 2. Material and Methods

### 2.1. HEI-OC1 Cell Culture

The HEI-OC1 cell line is extremely sensitive to ototoxic drugs, expresses several molecular markers which are characteristic of organ of Corti sensory cells [23], and therefore the HEI-OC1 cell line can be a useful study model of ototoxic drugs. The cells were maintained in high-glucose Dulbecco’s modified eagle’s medium (Gibco BRL, Grand Island, NY, USA) containing 10% fetal bovine serum (Gibco BRL, Grand Island, NY, USA) without antibiotics at 33 °C and 10% CO_2_ in air.

### 2.2. Cell Viability after Exposure to Cisplatin and Metformin

Cell viability was measured using a cell counting kit-8 (Dojindo Laboratories, Kumamoto, Japan). HEI-OC1 cells were seeded in 48-well plates, with 2 × 10^4^ cells in each well. The following day, the cells in metformin group and metformin/cisplatin group were treated with 1 mM of metformin. After 24 h, 15 μM of cisplatin which concentration is known to be result in 50% of cell viability in our experiments, were added to cisplatin group and metformin/cisplatin group. After 48 h, the CCK-8 solution was added to each well, and the plates were incubated for 30 min at 33 °C. The optical density was measured at 450 nm using a microplate reader (Spectra Max plus 384; Molecular devices, Sunnyvale, CA, USA).

### 2.3. Measurement of Caspase-8, Caspase-9 and Caspase-3 Activity at Different Time Points

To identify the effect of metformin on cisplatin-induced cell death at different time points, we divided cells in to 6 h, 12 h, 24 h, and 48 h groups. The cells in metformin/cisplatin group were treated with 1 mM of metformin. After 24 h, 15 μM of cisplatin were added to cisplatin group and metformin/cisplatin group. Auditory cell line lysates were obtained after 6 h, 12 h, 24 h and 48 h. 

The enzymatic activity of caspase-8, -9, and -3 was assayed with the fluorometric assay kit (K112, K118, K105; Biovision, Milpitas, California, USA) according to the manufacturer’s protocol. Auditory cell line lysate was prepared in a lysis buffer on ice for 10 min. After measuring the amount of protein, 50 ug of each sample was used. The sample was reacted with each substrate (LEHD-AFC, IETD-AFC, DEVD-AFC) for 2 h at 37 °C. The plates were read by microplate reader (Spectra Max, Molecular Devices, Sunnyvale, CA, USA) at a 400 nm excitation filter and a 505 nm emission filter. We obtained the results by performing the experiment of 5 times.

### 2.4. RNA Sample Preparation

To identify the effect of metformin on cisplatin-induced cell death and gene expression changes at different time points. HEI-OC1 cells were treated with 15 μM cisplatin, 1 mM metformin for 6 h, 12 h, 24 h, and 48 h and harvested. Their total RNA was extracted using RNasy plus mini kit (QIAGEN, Germany), according to the manufacturer’s instructions. RNA purity and integrity were evaluated by ND-1000 Spectrophotometer (Wilmington, NC, USA), Agilent 2100 Bioanalyzer (Agilent Technologies, Palo Alto, CA, USA). The RNA samples were used for mRNA microarray, and qRT-PCR experiments.

### 2.5. mRNA MicroArray Analysis

The Affymetrix Whole transcript Expression array process was executed according to the manufacturer’s protocol (GeneChip Whole Transcript PLUS reagent Kit), Applied Biosystems, Waltham, MA, USA). cDNA was synthesized using the GeneChip WT (Whole Transcript). Amplification kit as described by the manufacturer. The sense cDNA was then fragmented and biotin-labeled with TdT (terminal deoxynucleotidyl transferase) using the GeneChip WT Terminal labeling kit. Approximately 5.5 μg of labeled DNA target was hybridized to the Affymetrix GeneChip Mouse 2.0 ST Array at 45 °C for 16 h. Hybridized arrays were washed and stained on a GeneChip Fluidics Station 450 and scanned on a GCS3000 Scanner (Affymetrix). Signal values were computed using the Affymetrix^®^ GeneChip™ Command Console software.

### 2.6. Data Analysis

Raw data were extracted automatically in Affymetrix data extraction protocol using the software provided by Affymetrix GeneChip^®^ Command Console^®^ Software (AGCC). After importing CEL files, the data were summarized and normalized with robust multi-average (RMA) method implemented in Affymetrix^®^ Expression Console™ Software (EC). We exported the result with gene level RMA analysis and performed the differentially expressed gene (DEG) analysis.

Statistical significance of the expression data was determined using fold change. We investigated gene groups satisfying a FC ≥ 1.5 and *p* < 0.05 and used statistical methods to compare gene expression among the groups. For a DEG set, Hierarchical cluster analysis was performed using complete linkage and Euclidean distance as a measure of similarity.

Gene-Enrichment and Functional Annotation analysis for significant probe list was performed using KEGG (www.genome.jp/kegg/ accessed on 23 December 2022) and Gene Ontology (www.geneontology.org/ accessed on 23 December 2022). All data analysis and visualization of differentially expressed genes was conducted using R 3.1.2 (www.r-project.org accessed on 23 December 2022), and Morpheus (https://software.broadinstitute.org/morpheus accessed on 23 December 2022). To facilitate biological interpretation, we performed network analysis among the genes that were significantly expressed oppositely in response to cisplatin and the application of metformin (STRING; https://string-db.org accessed on 23 December 2022).

### 2.7. Quantitative Real-Time PCR

To confirm the mRNA expression levels of cisplatin-induced gene, total RNA was extracted from them using a Rneasy plus Mini Kit (Cat. No. 74134, Qiagen Inc., Germantown, MD, USA), cDNAs were synthesized from the total RNAs using an iScript cDNA Synthesis Kit (Bio-Rad, Hercules, CA, USA) according to the manufacturer’s protocol. Real-time PCR was performed using an iQ SYBR-Green supermix Bio-Rad, Hercules, CA, USA). The fold change of gene expression was calculated using the 2−ΔΔCt method. We obtained the results by performing the experiment for 5 times. 

Primers used for real-time PCR were as follows: CSF2 forward, 5′-atgcctgtcacgttgaatga-3′; reverse, 5′-ccgtagaccctgctcgaata-3′, FOS forward, 5′-gtccggttccttctatgcag-3′; reverse, 5′-taagtagtgcagcccggagt-3′, ATF3 forward, 5′-tgccaagtgtcgaaacaaga-3′; reverse, 5′-ccttcagctcagcattcaca-3′, JUN forward, 5′-agaggaagcgcatgaggaac-3′; reverse, 5′-ctgttccctgagcatgttgg-3′, ASK1 forward, 5′-gctcaagtcccagcccatag-3′; reverse, 5′-ctctcagccagccaggaagt -3′, TXNIP forward, 5′- taccccagaagctcctcctt-3′; reverse, 5′-gggctgtcttgagagtcgtc-3′, TXN1 forward, 5′-gcccttcttccattccctct-3′; reverse, 5′-aaggtcggcagcatttgact-3′, TXN2 forward, 5′-agaagatggtcgccaagcag-3′; reverse, 5′-ctggtcctcgtccttgatcc-3′, IL-6 forward, 5′-cacggccttccctacttcac-3′; reverse, 5′-ccacgatttcccagagaaca-3′, Nfkb forward, 5′-tctcaaagcagcaggagcag-3′; reverse, 5′-ggcaccactccctcatcttc-3′, TNF-α forward, 5′-agccgatgggttgtaccttg-3′; reverse, 5′-cggcagagaggaggttgact-3′, TP53 forward, 5′-acgggacagctttgaggttc-3′; reverse, 5′-gcagttcagggcaaaggact-3′, GAPDH forward, 5′-acccagaagactgtggatgg-3′; reverse, 5′-acacattgggggtaggaaca-3′. GAPDH was used as the reference gene. 

### 2.8. Statistical Analysis 

All values are represented as mean ± SD. For data analysis, we used the SPSS 23.0 statistical program. For the comparison of multiple groups in the caspase activities and qRT-PCR, ANOVA was used. A *p* value of <0.05 was considered statistically significant. For multiple comparisons, false discovery rate was used.

## 3. Results 

### 3.1. Metformin Reduced Apoptosis and Increased Cell Survival 

**Figure 1:**
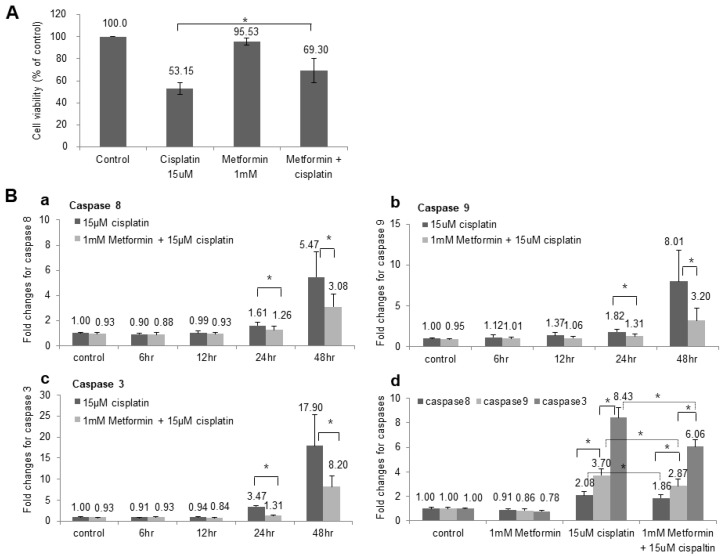
**Change of Cell viability and Caspase activity after metformin pretreatment.** (**A**) The cell viability was 53.15% when HEI-OC1 cells were exposed to cisplatin for 48 h. However, the cell viability increased to 69.30% when HEI-OC1 was pretreated with metformin for 24 h prior to application of cisplatin. (**B**) (**a**) Caspase 8 activity started to increase at 24 h (1.61-fold change) after the cisplatin application and significantly increased after 48 h (5.47-fold change). Metformin pretreatment reduced the activity of caspase 8 in both 24 h (1.26-fold change) and 48 h (3.08-fold change) and these were statistically significant. * *p* < 0.05. (**b**) Caspase 9 activity was increased to 1.82-fold change at 24 h and 8.01-fold change at 48 h after the cisplatin application. Metformin pretreatment reduced the activity of caspase 9 in both 24 h (1.31-fold change) and 48 h (3.20-fold change) and these were statistically significant. (**c**) Caspase 3 activity was increased to 3.47-fold change at 24 h and 17.90-fold change at 48 h after the cisplatin application. Metformin pretreatment reduced the activity of caspase 3 in both 24 h (1.31-fold change) and 48 h (8.20-fold change) and these were statistically significant. (**d**) When we compared the activity level of caspase 8, 9 and 3, they were increased to 2.08-, 3.70- and 8.43-fold change, respectively, after application of cisplatin for 48 h. The activity level of all three caspases were decreased to 1.86-, 2.87-, and 6.06-fold change after the pretreatment with metformin for 24 h (n = 5).

We analyzed the toxicity of cisplatin and the effect of metformin pretreatment in HEI-OC1 cell line. The cell viability was 53.15% in cisplatin group, but the cell viability increased to 69.30% when HEI-OC1 was pre-treated with metformin (Figure 1A), and the results were consistent with previous study [22]. In our previous study the pretreatment with metformin decreased apoptosis, reduced ROS production and lowered intracellular calcium concentration which were induced by cisplatin. 

We measured the caspase 8, 9 and 3 in time-dependent manner, and caspases started to increase at 24 h after the cisplatin application and significantly increased after 48 h. Metformin pretreatment reduced the activity of caspase 8, 9 and 3 in both 24 h and 48 h and these were statistically significant (Figure 1B). Additionally, when we compared the activity of caspase 8, 9 and 3, caspase 3 increased the most after cisplatin application followed by caspase 9 and 8 (Figure 1Bd). The fold changes of all three caspases were decreased after the pretreatment with metformin which indicates that metformin prevented cisplatin-induced apoptosis in both external and internal apoptotic pathway. 

### 3.2. Cisplatin- and Metformin-Related Gene Expression Profile in HEI-OC1

**Figure 2:**
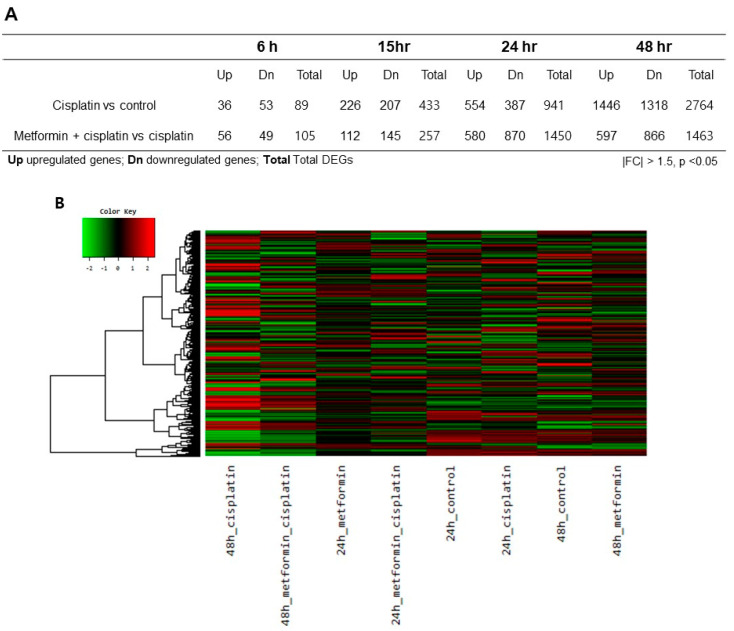

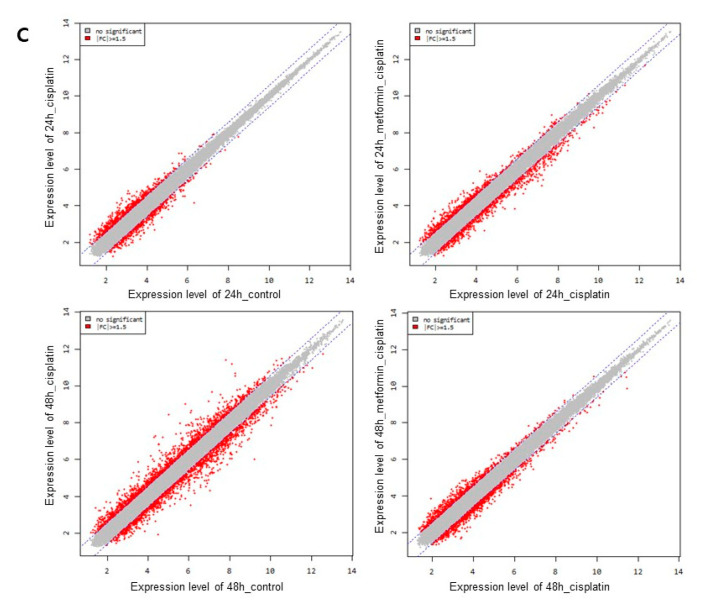
**Heatmap and volcano plots of RNA sequencing results.** (**A**). A total of 89 genes were differentially expressed with |fold change| > 1.5 (*p* < 0.05) after 6 h, a total of 433 genes were differentially expressed after 15 h, a total of 941 genes were differentially expressed after 24 h, and a total of 2,764 genes were differentially expressed after 48 h in cisplatin group. When metformin was pretreated for 24 h prior to application of cisplatin, a total of 105 genes were differentially expressed with |fold change| > 1.5 (*p* < 0.05) after 6 h of cisplatin exposure, a total of 257 genes were differentially expressed after 15 h, a total of 1,450 genes were differentially expressed after 24 h, and a total of 1,463 genes were differentially expressed after 48 h. (**B**). In the heatmap, the DEGs between the various selected biological conditions were analyzed (|fold change| > 1.5 and *p* < 0.05). Red indicates transcripts with high expression, and green indicates transcripts with low expression. **C.** Plots of expression level were drawn for comparisons between the control and cisplatin group, the metformin pretreated group and cisplatin group.

To determine whether gene expression is altered in response to exposure to cisplatin and metformin pretreatment, we identified the gene expression after cisplatin exposure according to different timelines. A total of 89 genes were differentially expressed with |fold change| > 1.5 (*p* < 0.05) after 6 h, a total of 433 genes were differentially expressed after 15 h, a total of 941 genes were differentially expressed after 24 h, and a total of 2764 genes were differentially expressed after 48 h in cisplatin group (Figure 2A). Then, we identified the gene expression after cisplatin exposure in metformin pretreated groups according to different timelines. A total of 105 genes were differentially expressed with |fold change| > 1.5 (*p* < 0.05) after 6 h of cisplatin exposure, a total of 257 genes were differentially expressed after 15 h, a total of 1450 genes were differentially expressed after 24 h, and a total of 1463 genes were differentially expressed after 48 h (Figure 2A). Since the expression level of caspases were significant after 24 h and 48 h, differentially expressed genes (DEGs) of 24 h and 48 h were chosen for further analysis. 

To identify the differentially expressed genes (DEGs) between groups, we generated a heat map of the expression values of the selected DEGs comparing across genes and samples (|fold change| > 1.5 and *p* < 0.05). The DEGs between the various selected biological conditions (Figure 2B). Additionally, plots of expression level were drawn for comparisons between the control and cisplatin group; the metformin pretreated group and cisplatin group (Figure 2C). Cisplatin induced significant changes in gene expression after 24 h and 48 h exposure; metformin pretreatments also induced significant changes in gene expression. 

KEGG pathway analysis revealed that cisplatin caused a change in the expression of genes involved in microRNAs in cancer, retinol metabolism, metabolic pathways in 24 h cisplatin group and metabolic pathways, microRNAs in cancer, PI3K-Akt signaling pathway, transcriptional misregulation in cancer, MAPK signaling pathway, pathways in cancer, axon guidance, proteoglycans in cancer, Rap1 signaling pathway, focal adhesion, p53 signaling pathway, cytokine–cytokine receptor interaction, TNF signaling pathway, endocytosis, FoxO signaling pathway and so on in 48 h cisplatin group (Tables S1 and S2). When metformin was pretreated, cisplatin caused a change in the expression of genes involved in transcriptional misregulation in cancer, microRNAs in cancer, PI3K-Akt signaling pathway, axon guidance, Rap1 signaling pathway, serotonergic synapse, focal adhesion, cytokine–cytokine receptor interaction, inflammatory mediator regulation of TRP channel, serotonergic synapse, metabolic pathways, p53 signaling pathway, pathways in cancer, tight junction and so on in 24 h metformin-pretreated group (Table 1, Table S3). Additionally, when metformin was pretreated, cisplatin caused a change in the expression of genes involved in microRNAs in cancer, cytokine–cytokine receptor interaction, phagosome, osteoclast differentiation, TNF signaling pathway in 48 h metformin-pretreated group (Table 2, Table S4). 

**Figure 3:**
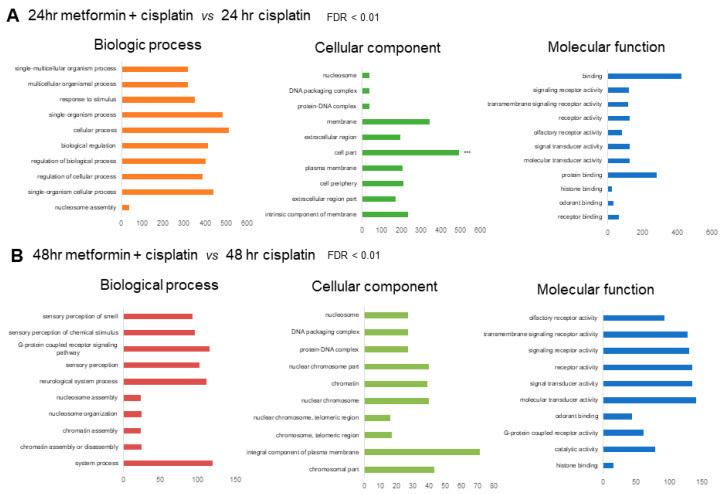
(**A**). GO annotation of predicted targets in 24 h metformin-pretreated group compared to the 24 h cisplatin group. The top 10 most enriched GO terms are listed in terms for biological process, cellular component, and molecular function based on *p*-values. (**B**). GO annotation of predicted targets in 48 h metformin-pretreated group compared to the 48 h cisplatin group. The top 10 most enriched GO terms are listed in terms for biological process, cellular component, and molecular function based on FDR <0.01.

For the 24 h metformin-pretreated group, the GO annotations of the predicted targets enriched among the 1450 genes that were mappable to DAVID, respectively, were selected according to a |fold-change| ≥ 1.5 and *p*-value ≤ 0.05 compared to the 24 h cisplatin group (Figure 3A). For the 48 h metformin-pretreated group, the GO annotations of the predicted targets enriched among the 1463 genes that were mappable to DAVID, respectively, were selected according to a |fold-change| ≥ 1.5 and *p*-value < 0.05 compared to the 48 h cisplatin group (Figure 3B). The functional annotation was categorized into biologic processes, cellular components and molecular functions, and only the top 10 GO terms showing the smallest *p*-values were considered.

Among the genes affected in the 24 h cisplatin group, the top 20 upregulated genes included CYP2c38, Nlrp9c, and Cyp4a12 which are related to inflammation; Gzmf, and Gzme which are related to cell death; krt6b and Pcdhb8 which are related to cell adhesion. The downregulated genes included Gsdmc3 which is related to membrane permeabilization and pyroptosis; and Tlr8 which is related to innate and adaptive immunity (Table 3). Among the genes affected in the 24 h metformin-pretreated group, the top 20 upregulated genes included Cdkn1a, Btg2, Txnip, and Ccne1 which are related to cell cycle; Akr1c13 and Txnip which are related to oxidative stress; Trav6-3 which are related to immune response; S100a7a involved in inflammatory response, Trp53inp1involved in autophagic cell death. The top 20 downregulated genes included Cdh18 and Magi1 which are involved in cell–cell adhesion; Gpc6, Plxna2, and Ptprg which are related with cell migration; Camk1d involved in apoptosis; Dis3l2 and Cdk14 involved in cell cycle; Tbc1d5 involved in autophagy; and Prkca which is related with multiple biologic process such as cell adhesion, cell migration, apoptosis signaling, proliferation, inflammation (Table 4).

Among the genes affected in the 48 h cisplatin group, the upregulated genes included Txnip, Btg2, and Cdkn1a which are related to cell cycle; Txnip which are related to oxidative stress; Trp53inp1 involved in autophagic cell death; Eda2r, Mdm2 and Fas which are involved in apoptotic cell death; Ptgs2 and S100a7a involved in inflammatory response; Fos involved in cell proliferation and differentiation; Tnfsf18 involved in T-cell responses; Egr1 which is related with the regulation of cell survival, proliferation and cell death. The top 20 downregulated genes included Ptn and Gas1 which are involved cell growth; Cdh18 and Magi1 which are involved in cell–cell adhesion (Table 5). Among the genes affected in the 48 h metformin-pretreated group, the top 20 upregulated genes included Cdsn involved in cell–cell adhesion; Ear1 and Mpeg1 involved in immune response; Eda related with cell death; Cd27 which is involved in apoptosis. The top 20 downregulated genes included Csf2, cytokine that stimulates the growth and differentiation of hematopoietic precursor cells from various lineages; Tnfsf18 which is involved in T-cell response; Fos and Egr1 which are related with cell proliferation and death, Btg2 involved with cell cycle regulation; Fos and Nr4a2 which are involved in cellular response to oxidative stress (Table 6).

**Figure 4:**
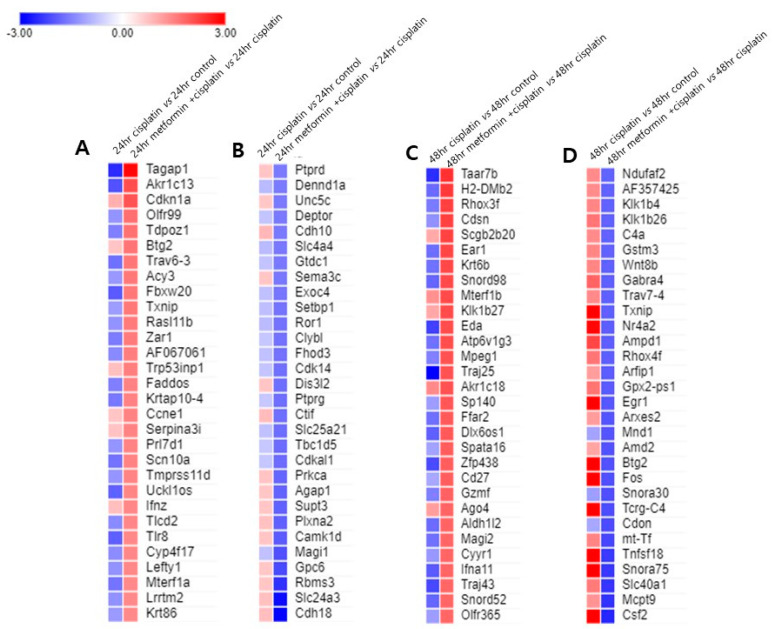
**Metformin pretreatment-triggered gene expression changes compared to cisplatin group.** (**A**). The expression level of top 30 upregulated genes in 24 h metformin-pretreated group were compared with those of 24 h cisplatin group. Pretreatment of metformin for 24 h increased the expression of Tagap1, Akr1c13, Trav6-3, Fbxw20, Scn10a, Uckl1os, Tlr8, and Mterf1a more than |fold-change| ≥ 1.5 while significantly decreasing same genes with |fold-change| ≥ 1.5 in cisplatin alone group. (**B**). The expression level of top 30 downregulated genes in 24 h metformin-pretreated group were not in accord with elevated genes in 24 h cisplatin group. (**C**). Pretreatment of metformin for 24 h and then exposure to cisplatin for 48 h (48 h metformin-pretreated group) increased the expression of Taar7b, H2-DMb2, Rhox3f, Ear1, Krt6b, Snord98, Eda, Atp6v1g3, Mpeg1, Traj25, Ffar2, Dlx6os1, Zfp438, Gzmf, Aldh1l2, Magi2, Ifna11, Tfaj43, and Snord52 more than |fold-change| ≥ 1.5 while significantly decreasing same genes with |fold-change| ≥ 1.5 in 48 h cisplatin group. (**D**). Likewise, in 48 h metformin-pretreated group, the expression of Csf2, Slc40a1, Snora75, Tnfsf18, Tcrg-C4, Fos, Btg2, Egr1, Gpx2-ps1, Rhox4f, Ampd1, Nr4a2, Txnip, Gabra4 and Klk1b26 decreased more than |fold-change| ≥ 1.5 while significantly increasing same genes with |fold-change| ≥ 1.5 in the 48 h cisplatin group. Red indicates transcripts with high expression, and blue indicates transcripts with low expression.

To identify whether metformin pretreatment caused the gene expression opposite to the cisplatin alone treated group, we’ve selected top 30 upregulated and downregulated genes in both 24 h and 48 h metformin pretreatment groups and evaluated the gene expression level of the 24 h and 48 h cisplatin groups and drawn heat maps (Figure 4). Pretreatment of metformin for 24 h increased the expression of Tagap1, Akr1c13, Trav6-3, Fbxw20, Scn10a, Uckl1os, Tlr8, and Mterf1a more than |fold-change| ≥ 1.5 while significantly decreasing same genes with |fold-change| ≥ 1.5 in cisplatin alone group (Figure 4A). However, top 30 downregulated genes in 24 h metformin pretreatment group were not in accord with elevated genes in cisplatin alone group (Figure 4B). Pretreatment of metformin for 48 h increased the expression of Taar7b, H2-DMb2, Rhox3f, Ear1, Krt6b, Snord98, Eda, Atp6v1g3, Mpeg1, Traj25, Ffar2, Dlx6os1, Zfp438, Gzmf, Aldh1l2, Magi2, Ifna11, Tfaj43, and Snord52 more than |fold-change| ≥ 1.5 while significantly decreasing same genes with |fold-change| ≥ 1.5 in 48 h cisplatin group (Figure 4C). Likewise, pretreatment of metformin for 48 h decreased the expression of Csf2, Slc40a1, Snora75, Tnfsf18, Tcrg-C4, Fos, Btg2, Egr1, Gpx2-ps1, Rhox4f, Ampd1, Nr4a2, Txnip, Gabra4 and Klk1b26 more than |fold-change| ≥ 1.5 while significantly increasing same genes with |fold-change| ≥ 1.5 in the 48 h cisplatin group (Figure 4D).

### 3.3. Network Analysis and qRT-PCR Expression Levels of the Potential Biomarkers

**Figure 5:**
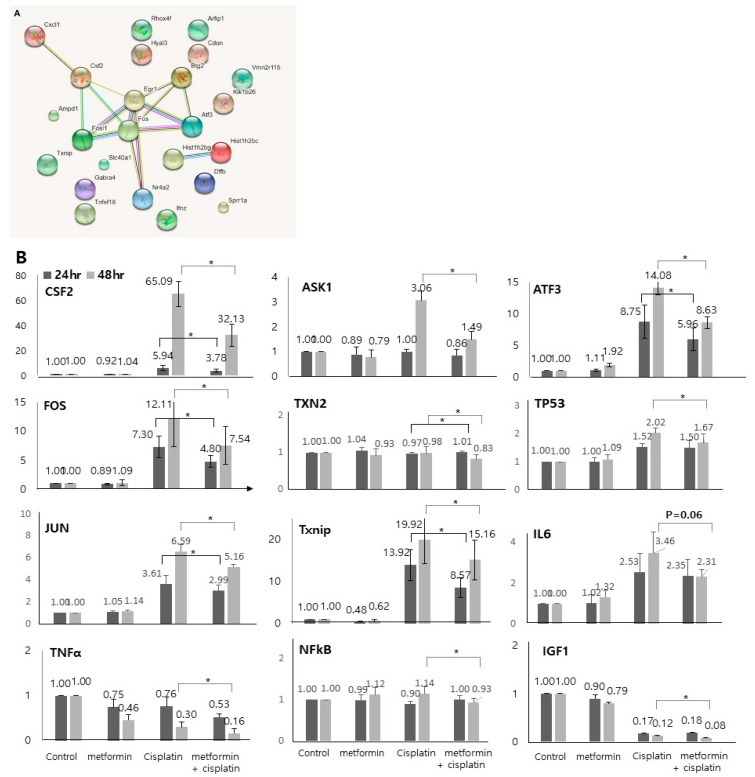
**Network analysis and qRT-PCR expression levels of the potential biomarkers.** (**A**). The network analysis was performed among genes that were significantly expressed oppositely in response to cisplatin and the application of metformin (48 cisplatin group vs. 48 h metformin-pretreated group). Various genes such as Fos, Fosl1, Csf2, Cxcl1, Egr1, Btg2, Atf3, and Nr4a2 were identified to be related to each other. (**B**) 48 h cisplatin exposure-induced and metformin-pretreated prior to 48 h cisplatin exposure-induced genes validated by qRT-PCR. In accordance with the microarray results, the expression levels of CSF2, FOS, JUN, TNFα, NFκB, Txnip, ASK1, TXN2, ATF3, TP53, and IGF were found to be decreased after application of metformin (*p* < 0.05). The expression levels of IL6 decreased after application of metformin but it was not statistically significant (*p* = 0.06). Solid lines show the statistics between 24 h cisplatin group and 24 metformin-pretreated group. Dotted lines show the statistics between 48 h cisplatin group and 24 metformin-pretreated group (n = 5).

The molecular signaling networks among the genes that were differentially expressed in response to application of cisplatin and metformin were analyzed to predict the relevant molecular pathways. The analysis was performed among genes that were significantly expressed oppositely in response to cisplatin and the application of metformin (Table S5, Table 7). Various genes such as Fos, Fosl1, Csf2, Cxcl1, Egr1, Btg2, Atf3, and Nr4a2 were identified to be related to each other in 48 h groups (Figure 5A).

Based on the gene expression and network analyses, we investigated several genes (CSF2, FOS, JUN, TNFα, NFκB, Txnip, ASK1, TXN2, ATF3, TP53, IL6, and IGF1) as metformin-related preventive biomarkers in cisplatin ototoxicity. The selection of key gens was performed based on the analysis of genes that were oppositely expressed in response to cisplatin and the application of metformin (Table 7), and on the analysis of genes that were most up and downregulated after 48 h treatment with cisplatin with or without application of metformin (Table 5 and Table 6) and on well-known related genes. To validate the microarray results, we examined the expressed transcript levels by qRT-PCR. In accordance with the RNA sequencing results, the expression levels of CSF2 (5.94 vs. 3.78, *p* = 0.006 for 24 h; 65.09 vs. 32.13, *p* = 0.003 for 48 h), FOS (7.30 vs. 4.80, *p* = 0.005 for 24 h; 12.00 vs. 7.54, *p* = 0.000 for 48 h), JUN (3.61 vs. 2.99, *p* = 0.043 for 24 h; 6.59 vs. 5.16, *p* = 0.007 for 48 h), TNFα (0.30 vs. 0.16, *p* = 0.008 for 48 h), NFκB (1.14 vs. 0.93, *p* = 0.031 for 48 h), Txnip (13.92 vs. 8.57, *p* = 0.005 for 24 h; 19.92 vs. 15.16, *p* = 0.002 for 48 h), ASK1 (3.06 vs. 1.49, *p* = 0.000 for 48 h), TXN2 (0.97 vs. 1.01, *p* = 0.026 for 24 h; 0.98 vs. 0.83, *p* = 0.047 for 48 h), ATF3 (8.75 vs. 5.96, *p* = 0.006 for 24 h; 14.08 vs. 8.63, *p* = 0.000 for 48 h), TP53 (2.02 vs. 1.67, *p* = 0.021 for 48 h), and IGF (1.12 vs. 0.08, *p* = 0.035 for 48 h) were found to be decreased after application of metformin (Figure 5B). The expression levels of IL6 decreased after application of metformin but it was not statistically significant (3.46 vs. 2.31, *p* = 0.060 for 48 h).

## 4. Discussion

In this study, we evaluated the change of gene expression induced by cisplatin at several different time points. We identified that cisplatin application caused delayed gene expression changes, which means that as the exposure time to cisplatin lengthened, the number of affected genes increased. Genes which were upregulated or downregulated after 24 h exposure to cisplatin were genes that were related with inflammation (CYP2c38, Nlrp9c, and Cyp4a12), cell adhesion (krt6b and Pcdhb8), membrane permeabilization and pyroptosis (Gsdmc3), and cell death (Gzmf and Gzme). Exposure to cisplatin for 48 h altered genes that were related with cell cycle (Txnip, Btg2, and Cdkn1a), oxidative stress (Txnip), inflammatory process (Ptgs2 and S100a7a), cell proliferation and cell death (Fos, Egr1), autophagic cell death (Trp53inp1), and apoptotic cell death (Eda2, Mdm2 and Fas). These data support the cellular change at the gene level after the application of cisplatin.

There are several reports demonstrating the gene expression change in cisplatin-induced ototoxicity in HEI-OC1 cells using RNA sequencing analysis [24,25]. In a study evaluating the gene expression change after 30 h cisplatin exposure, downregulated DEGs were associated with several KEGG pathways such as ‘systemic lupus erythematosus’, ‘alcoholism’, ‘viral carcinogenesis’, ‘PI3K-Akt signaling pathway’, ‘Rap1 signaling pathway’, and ‘HIF-1 signaling pathway’; which are in accord with our data (Supplementary Table S2). They also reported that upregulated DEGs were significantly associated with autophagy, apoptosis-associated processes, response to DNA damage and cell cycle arrest; and demonstrated several genes such as phorbol-12-myristate-13-acetate-induced protein 1 (PMAIPI), Bcl-2 binding component 3 (BBC3), zinc finger matrin-type 3 (ZMAT3), p53-induced death domain protein 1 (PIDD1), B-cell translocation gene protein 2 (BTG2), thioredoxin-inter-acting protein (TXNIP), DNA damage induced apoptosis suppressor (DDIAS), cycle-dependent kinase inhibitor 1a (Cdkn1a), transformation related protein 53-induced nuclear protein 1 (Trp53inp1), Foxo3, and Fas; which are also similar to our results.

In our previous study, pretreatment with metformin prior to the application of cisplatin significantly decreased the late apoptosis in HEI-OCI cells [22]. Metformin attenuated the cisplatin-induced increase in ROS, inhibited the activation of caspase-3 and levels of poly-ADP-ribose polymerase (PARP) and prevented the cisplatin-induced elevation in intracellular calcium concentrations after 48 h exposure to cisplatin. In this study, we measured the caspase 8, 9 and 3 in time-dependent manner, and identified that caspases started to increase at 24 h after the cisplatin application and significantly increased after 48 h. Metformin pretreatment reduced the expression of caspase 8, 9 and 3 in both 24 h and 48 h, indicating that metformin is involved in both intrinsic and extrinsic apoptotic pathways [26].

We identified several genes (CSF2, FOS, JUN, TNFα, NFκB, Txnip, ASK1, TXN2, ATF3, TP53, IL6, and IGF1) which are related with the preventive effects of metformin in cisplatin ototoxicity based on the gene expression and network analyses. Among these genes, thioredoxin-interacting protein (Txnip) is reported to have relation with cell apoptosis and inflammation [27,28]. The primary role of Txnip is inhibition of thioredoxin (TRX), an important redox protein which controls levels of reactive oxygen species, and the inhibition of TRX by Txnip promotes inflammation and increases levels of ROS [29,30]. Additionally, the redox-regulated apoptosis-signal kinase (ASK1), a member of the mitogen- activated protein kinase family, is considered as an important link between cellular stress and innate immunity [31,32]. ASK1 is usually bound to mitochondrial thioredoxin (TRX2) under normal conditions, but, during stress and following Txnip translocation to the mitochondria, ASK1-TRX2 binding is interrupted and triggers an apoptotic signal cascade leading to cytochrome c release and caspase-3 cleavage, and apoptosis [33].

Metformin is known to exert direct immunomodulatory effect on immune cells by AMPK induction and mTORC1 inhibition. The mTOR is subcategorized into two multiprotein complexes, mTOR complex 1 (mTORC1) and mTOR complex 2 (mTORC2), which could be differentiated based on their related protein [34]. mTORC1 is responsible for cell growth and proliferation in response to growth factors and nutrient while mTORC2 is insensitive to nutrients. The two main downstream targets of mTORC1 are p70S6 kinase (S6K) and elongation factor 4E binding protein (4E-BP1) [35]; although mTORC2 is relatively unknown, they are reported to be related with PI3K, and Akt [36].

There are relatively many studies regarding the role of mTOR in cancer, but it is not much known about the role of mTOR in inner ear. Recent study reported that a low dose intraperitoneal injection of sirolimus (mTOR inhibitor) attenuated age-related hearing loss by decreasing the mTORC1, while high dose of sirolimus caused severe hearing loss by decreasing mTORC2/Akt. They also reported that mTORC2/Akt is involved in the regulation of hair cell survival in the cisplatin exposure condition, suggesting that the therapeutic activation of mTORC2 in conjunction with decreasing mTORC1 might represent a promising and effective strategy in preventing hearing loss [37].

In pancreatic β cell, mTOR is reported to associate with the carbohydrate-response element–binding protein (ChREBP)–Max-like protein complex and inhibit its transcriptional activity, leading to decreased expression of TXNIP, a potent inducer of pancreatic β cell death and oxidative stress. Meanwhile, mTOR inhibitor or mTOR deficiency enhances transcriptional activity and activates Txnip expression [38]. Additionally, mTOR and HDAC inhibitors converge on the TXNIP/thioredoxin pathway and cause oxidative stress and apoptosis in esophageal cancer [39].

However, in our study, we identified that metformin, which is mTOR inhibitor, decreased the expression level of Txnip compared to the level evoked by cisplatin. Cisplatin increased the expression of Txnip in 24 h and 48 h exposure group, but pretreatment with metformin reduced the Txnip expression in HEI-OC1 cells. ASK1 was also elevated after 48 h application of cisplatin but when HEI-OC1 cells were pretreated with metformin prior to application of cisplatin, the level of both Txnip and ASK1 reduced. Previous studies also demonstrated that metformin significantly reduced Txnip mRNA and protein expression [40]. Although, our experiment was performed on HEI-OC1 cell line and further validation studies are required in the future, our observation suggests that metformin prevented the inhibitory effect of Txnip which primary role is to inhibit TRX, lowered levels of ROS, and reduced apoptotic cascade signals by decreasing ASK1 expression which would lead to caspase-3 cleavage and apoptosis.

## 5. Conclusions

In this study, we evaluated the change of gene expression induced by cisplatin at several different time points, and the alteration of gene expression according to pretreatment with metformin in HEI-OC1 cells through microarray analysis. We have identified potential molecular modulator genes (CSF2, FOS, JUN, TNFα, NFκB, Txnip, ASK1, TXN2, ATF3, TP53, IL6, and IGF1) that might be related with the preventive role of metformin in the cisplatin ototoxicity. Additionally, our observation suggests that metformin would prevent the expression of Txnip and ASK1 and thus lowers ROS levels, and reduces apoptotic cascade signals in cisplatin-induced ototoxicity.

## Figures and Tables

**Table 1:** Analysis of enrichment of KEGG pathways and involved genes in 24 h metformin-pretreated group.

MapName	SigGenes	Bonferroni	FDR
Transcriptional misregulation in cancer	Supt3, Runx1, Cd14, Cdkn1a, Eya1, Igf1, Igf1r, Mdm2, Pbx1, Cdk14, Tgfbr2, Gria3, Gm12657, Mllt3,Hist2h3b//Hist1h3e//Hist1h3b//Hist1h3d//Hist1h3c//Hist1h3f//Hist2h3c2//Hist2h3c1, Hist1h3d, Hist2h3b//Hist1h3e//Hist1h3b//Hist1h3d//Hist1h3c//Hist1h3f//Hist2h3c2//Hist2h3c1, Hist1h3a//Hist1h3i//Hist1h3h//Hist1h3g Hist2h3b//Hist1h3e//Hist1h3b//Hist1h3d//Hist1h3c//Hist1h3f//Hist2h3c2//Hist2h3c1Hist1h3a//Hist1h3i//Hist1h3h//Hist1h3g, Hist2h3b//Hist1h3e//Hist1h3b//Hist1h3d//Hist1h3c//Hist1h3f//Hist2h3c2//Hist2h3c1	3.134 × 10^−19^	6.268 × 10^−20^
MicroRNAs in cancer	Bcl2, Ccne1, Cdk6, Cdkn1a, Socs1, Mdm2, Mmp16, Pdgfb, Pdgfrb, Prkca, Prkce, Thbs1, Tnr, Zfpm2, Mir10b, Mir23a, Mir29a, Mir30c-1, Mir99a, Mirlet7b, Mir28a, Mir31, Mir335, Mir194-2	5.4124 × 10^−13^	9.0207 × 10^−14^
PI3K-Akt signaling pathway	Itga1, Angpt1, Bcl2, Ccne1, Cdk6, Cdkn1a, Col11a1, Efna5, Fgf7, Ghr, Igf1, Igf1r, Mdm2, Pdgfb, Pdgfrb, Prkca, Thbs1, Thbs2, Tnr, Col5a3, Pdgfd	2.5104 × 10^−8^	3.5863 × 10^−9^
Axon guidance	Bcl2, Ccne1, Cdk6, Cdkn1a, Socs1, Mdm2, Mmp16, Pdgfb, Pdgfrb, Prkca, Prkce, Thbs1, Tnr, Zfpm2, Mir10b, Mir23a, Mir29a, Mir30c-1, Mir99a, Mirlet7b, Mir28a, Mir31, Mir335, Mir194-2	7.823 × 10^−8^	9.7787 × 10^−9^
Rap1 signaling pathway	Angpt1, Efna5, Fgf7, Magi1, Igf1, Igf1r, Pdgfb, Pdgfrb, Prkca, Plcb4, Thbs1, Map2k6, Pdgfd, Pard6g, Pard3	3.0193 × 10^−6^	3.3548 × 10^−7^
Focal adhesion	Itga1, Bcl2, Col11a1, Igf1, Igf1r, Pdgfb, Pdgfrb, Prkca, Thbs1, Thbs2, Tnr, Dock1, Col5a3, Pdgfd	1.6449 × 10^−5^	1.6449 × 10^−6^
Cytokine–cytokine receptor interaction	Eda, Ghr, Il10ra, Il11, Lepr, Pdgfb, Pdgfrb, Ccl5, Cxcl5, Cxcl12, Tgfbr2, Tnfsf9, Tnfsf18, Ppbp, Pdgfd	3.8745 × 10^−5^	3.5223 × 10^−6^
Inflammatory mediator regulation of TRP channels	Cyp2j11, Cyp2c38, Igf1, Itpr1, Itpr2, Prkca, Prkce Plcb4, Map2k6, Cyp4a12a, Cyp2c55	7.3159 × 10^−5^	6.0966 × 10^−6^
Serotonergic synapse	Cyp2j11, Cacna1c, Cyp2c38, Cyp2d10, Htr5b, Itpr1, Itpr2, Prkca, Plcb4, Dusp1, Cyp2c55	0.00011611	8.2939 × 10^−6^
Metabolic pathways	Cyp2j11, Me3, Pcca, Adk, Bst1, Cbr1, Cyp26a1, Cyp2c38, Nat3, Plcb4, Pld1, Dhrs3, St3gal3, Suclg2, Itpk1, Gmds, Ak5, Galnt18, B3galt1, Galnt13, Cyp4a12a, Tpk1, Dgkh, Ndst4, Lpin2, Ppap2b, Bdh1, Cyp2c55, Nmnat3, Gbe1, Chsy3	0.00027672	1.6277 × 10^−5^
p53 signaling pathway	Ccne1, Ccng2, Cdk6, Cdkn1a, Gadd45a, Igf1, Mdm2, Thbs1	0.00077208	3.7439 × 10^−5^
Pathways in cancer	Bcl2, Bmp4, Runx1, Ccne1, Cdk6, Cdkn1a, Fgf7, Igf1, Igf1r, Mdm2, Pdgfb, Pdgfrb, Prkca, Plcb4, Cxcl12, Tgfbr2	0.00078621	3.7439 × 10^−5^
Tight junction	Cask, Cldn1, Magi1, Myh4, Prkca, Prkce, Exoc4, Mpp5, Pard6g, Pard3	0.00135494	6.1588 × 10^−5^
MAPK signaling pathway	Cacna1c, Cacna2d1, Cacnb3, Cd14, Gadd45a, Fgf7, Pdgfb, Pdgfrb, Prkca, Dusp1, Tgfbr2, Map2k6	0.00463177	0.00019299
Vascular smooth muscle contraction	Adra1d, Cacna1c, Itpr1, Itpr2, Prkca, Prkce, Plcb4, Cyp4a12a, Calcrl	0.00566393	0.00021784
Choline metabolism in cancer	Pdgfb, Pdgfrb, Prkca, Pld1, Slc22a5, Dgkh, Ppap2b, Pdgfd	0.00931772	0.0003451
Ras signaling pathway	Angpt1, Efna5, Fgf7, Igf1, Igf1r, Pdgfb, Pdgfrb, Prkca, Pld1, Rasal1, Pdgfd	0.01057243	0.00037759
Neuroactive ligand-receptor interaction	Adra1d, C3ar1, Galr1, Ghr, Gpr50, Htr5b, Lepr, Gria3, Calcrl, 2210010C04Rik, 1810009J06Rik, Pard3	0.01189479	0.00041017
Oxytocin signaling pathway	Cacna1c, Cacna2d1, Cacnb3, Cdkn1a, Itpr1, Itpr2, Prkca, Plcb4, Camk1d	0.02295549	0.00076518
Gap junction	Itpr1, Itpr2, Pdgfb, Pdgfrb, Prkca, Plcb4, Pdgfd	0.03375284	0.0010888
GnRH signaling pathway	Cacna1c, Itpr1, Itpr2, Prkca, Plcb4, Pld1, Map2k6	0.03793504	0.00118547
Endocytosis	Asap1, H2-Q10, Igf1r, Mdm2, Pld1, Asap2, Gmds, Agap1, Nedd4l, Pard6g, Pard3	0.04708001	0.00138471
FoxO signaling pathway	Ccng2, Cdkn1a, Gadd45a Igf1, Igf1r, Mdm2, Gmds, Gabarapl	0.04959527	0.00141701
Calcium signaling pathway	Phkb, Adra1d, Cacna1c, Htr5b, Itpr1, Itpr2, Pdgfrb, Prkca, Plcb4	0.053559531	0.00148776
HIF-1 signaling pathway	Angpt1, Bcl2, Cdkn1a, Edn1, Igf1, Igf1r, Prkca	0.106382279	0.0028752
Glutamatergic synapse	Cacna1c, Itpr1, Itpr2, Prkca, Plcb4, Pld1, Gria3	0.145348043	0.00382495
Phosphatidylinositol signaling system	Itpr1, Itpr2, Prkca, Plcb4, Itpk1, Dgkh	0.202974495	0.00520447
Cell cycle	Ccne1, Cdk6, Cdkn1a, Gadd45a, Mad1l1, Mdm2, Prkdc	0.210708128	0.0052677
ECM-receptor interaction	Itga1, Col11a1, Thbs1, Thbs2, Tnr, Col5a3	0.273774372	0.00636685
Fc gamma R-mediated phagocytosis	Asap1, Prkca, Prkce, Pld1, Asap2, Ppap2b	0.273774372	0.00636685
TNF signaling pathway	Edn1, Ccl5, Map2k6, Cxcl3, Gm5431, Ripk3	0.719880182	0.01469143
Regulation of actin cytoskeleton	Itga1, Cd14, Fgf7, Pdgfb, Pdgfrb, Dock1, Diap2, Pdgfd	0.751289578	0.01502579
Cholinergic synapse	Bcl2, Cacna1c, Itpr1, Itpr2, Prkca, Plcb4	0.802839476	0.01574195
Renin secretion	Cacna1c, Clca1, Itpr1, Itpr2, Plcb4	0.905722389	0.01741774
cGMP-PKG signaling pathway	Cacna1c, Clca1, Itpr1, Itpr2, Plcb4	0.978637319	0.01774273
Regulation of autophagy	Ulk1, Gabarapl1, Atg10, Atg7	1	0.02257359
TGF-beta signaling pathway	Bmp4, Lefty1, Fst, Tgfbr2, Thbs1	1	0.02417471
Dopaminergic synapse	Cacna1c, Itpr1, Itpr2, Prkca, Plcb4, Gria3	1	0.02675152
Arachidonic acid metabolism	Cyp2j11, Cbr1, Cyp2c38, Cyp4a12a, Cyp2c55	1	0.02920426
Retinol metabolism	Cyp26a1, Cyp2c38, Dhrs3, Cyp4a12a, Cyp2c55	1	0.02920426
Chemokine signaling pathway	Cbr1, Cyp2c38, Gstm6, Nat3, Cyp2c55	1	0.03048876
Jak-STAT signaling pathway	Socs1, Ghr, Il10ra, Il11, Lepr, Spry1	1	0.04055011
Toll-like receptor signaling pathway	Cd14, Tlr8, Ly96, Ccl5, Map2k6	1	0.04089946

**Table 2:** Analysis of enrichment of KEGG pathways and involved genes in 48 h metformin-pretreated group.

MapName	Genes	Bonferroni	FDR
MicroRNAs in cancer	Mir101c, Ccng1, Pdgfra, Mir215, Mir30c-1, Mirlet7b, Mir107, Mir34a, Mir181b, Mir335, Mir129-2, Mirlet7c-2	0.00074855	0.00014971
Cytokine–cytokine receptor interaction	Csf2, Csf2rb, Eda, Cxcl1, Ifna11, Il9, Lepr, Pdgfra, Il23r, Cd27, Tnfsf18	0.00336159	0.00048023
Phagosome	Actg1, Coro1a, H2-DMb2, H2-Q1, Thbs3, Tfrc, Tuba3b//Tuba3a, Tuba3b//Tuba3a, Atp6v1g3	0.00605809	0.00067547
Osteoclast differentiation	LOC100038947, Fos, Fosb, Fosl1, Gab2, Jun, Junb, Stat2	0.00607925	0.00067547
TNF signaling pathway	Csf2, Fos, Cxcl1, Jun, Junb, Sele, Magi2	0.02605061	0.00260506
cAMP signaling pathway	Camk2a, Fos, Grin2a, Jun, Pld1, Atp1a3, Ffar2, Ghrl	0.091690159	0.00764085
Rap1 signaling pathway	Actg1, Angpt2, Grin2a, Klk1b4, Pdgfra, Dock4, Sipa1l2, Magi2	0.138968297	0.01029513
Jak-STAT signaling pathway	Csf2, Csf2rb, Ifna11, Il9, Lepr, Stat2, Il23r	0.144131818	0.01029513
B cell receptor signaling pathway	Cd22, Cr2, Fos, Jun, Ifitm1	0.338145688	0.02113411
Steroid hormone biosynthesis	Akr1c18, Cyp3a13, Hsd17b6, Cyp3a44, Cyp2d11	0.621540012	0.02971865
Metabolic pathways	Cth, Gpt2, Hyal3, Pygl, Cyp3a13, Pld1, Pycr1, Ampd1, Ggt5, Gulo, Lpcat2, Acsm5, Hsd17b6, Cyp3a44, Atp6v1g3, Aldh18a1, Tdo2, Dcxr, Pck2	0.817243672	0.03714744
Toll-like receptor signaling pathway	Cd80, Fos, Ifna11, Jun, Spp1	1	0.04339047
Cell adhesion molecules (CAMs)	Cd22, Cd80, H2-DMb2, H2-Q1, Sele, Cldn20	1	0.04339047

**Table 3:** List of top 20 up and downregulated genes in 24 h cisplatin group.

Gene Name	Gene Description	Fold Change	Gene Name	Gene Description	Fold Change
Cyp2c38	cytochrome P450, family 2, subfamily c, polypeptide 38	2.419	Tagap1	T cell activation GTPase activating protein 1	−2.400
Gzmf	granzyme F	2.417	Lpin2	lipin 2	−2.090
Nlrp9c	NLR family, pyrin domain containing 9C	2.238	Akr1c13	aldo-keto reductase family 1, member C13	−1.950
Krt6b	keratin 6B	2.151	Taar7d	trace amine-associated receptor 7D	−1.928
Scgb2b10	secretoglobin, family 2B, member 10	2.125	Gsdmc3	gasdermin C3	−1.918
Rex2	reduced expression 2	2.019	Hoxd13	homeobox D13	−1.901
Amy2b	amylase 2b	1.985	Cyp26a1	cytochrome P450, family 26, subfamily a, polypeptide 1	−1.864
Esp3	exocrine gland secreted peptide 3	1.961	Agbl2	ATP/GTP binding protein-like 2	−1.861
Ear1	eosinophil-associated, ribonuclease A family, member 1	1.918	Fbxw20	F-box and WD-40 domain protein 20	−1.852
Ipw	imprinted gene in the Prader-Willi syndrome region	1.853	Krtap6-2	keratin associated protein 6-2	−1.841
Trbj1-7	T cell receptor beta joining 1-7	1.850	Tnfrsf18	tumor necrosis factor receptor superfamily, member 18	−1.819
Gzme	granzyme E	1.824	Cmtm1	CKLF-like MARVEL transmembrane domain containing 1	−1.804
Cyp4a12a	cytochrome P450, family 4, subfamily a, polypeptide 12a	1.811	Trav14-1	T cell receptor alpha variable 14-1	−1.795
Traj24	T cell receptor alpha joining 24	1.806	Tlr8	Toll-like receptor 8	−1.791
Foxn4	forkhead box N4	1.7920	Sh2d1b2	SH2 domain protein 1B2	−1.783
Skint5	selection and upkeep of intraepithelial T cells 5	1.776	Svs3b	seminal vesicle secretory protein 3B	−1.767
Klra9	killer cell lectin-like receptor subfamily A, member 9	1.768	Uckl1os	uridine-cytidine kinase 1-like 1, opposite strand	−1.762
Adck1	aarF domain containing kinase 1	1.759	Abcd2	ATP-binding cassette, sub-family D (ALD), member 2	−1.750
Pcdhb8	protocadherin beta 8	1.736	Dsg1b	desmoglein 1 beta	−1.726
Aarsd1	alanyl-tRNA synthetase domain containing 1	1.707	Dpt	dermatopontin	−1.703

FDR < 0.01.

**Table 4:** List of top 20 up and downregulated genes in 24 h metformin-pretreated group.

Gene Name	Gene Description	Fold Change	Gene Name	Gene Description	Fold Change
Tagap1	T cell activation GTPase activating protein 1	3.31	Cdh18	cadherin 18	−4.34
Akr1c13	aldo-keto reductase family 1, member C13	2.49	Slc24a3	solute carrier family 24 (sodium/potassium/calcium exchanger), member 3	−4.15
Cdkn1a	cyclin-dependent kinase inhibitor 1A (P21)	2.43	Gpc6	glypican 6	−3.18
Tdpoz1	TD and POZ domain containing 1	2.06	Magi1	membrane associated guanylate kinase, WW and PDZ domain containing 1	−3.07
Btg2	B cell translocation gene 2, anti-proliferative	2.02	Camk1d	calcium/calmodulin-dependent protein kinase ID	−2.90
Trav6-3	T cell receptor alpha variable 6-3	2.00	Plxna2	plexin A2	−2.79
Acy3	aspartoacylase (aminoacylase) 3	1.99	Supt3	suppressor of Ty 3	−2.77
Fbxw20	F-box and WD-40 domain protein 20	1.97	Agap1	ArfGAP with GTPase domain, ankyrin repeat and PH domain 1	−2.75
Txnip	thioredoxin interacting protein	1.97	Prkca	protein kinase C, alpha	−2.72
Rasl11b	RAS-like, family 11, member B	1.95	Cdkal1	CDK5 regulatory subunit associated protein 1-like 1	−2.60
Zar1	zygote arrest 1	1.94	Tbc1d5	TBC1 domain family, member 5	−2.60
Trp53inp1	transformation related protein 53 inducible nuclear protein 1	1.90	Slc25a21	solute carrier family 25 (mitochondrial oxodicarboxylate carrier), member 21	−2.58
Faddos	Fas (TNFRSF6)-associated via death domain, opposite strand	1.86	Ctif	CBP80/20-dependent translation initiation factor	−2.56
Krtap10-4	keratin associated protein 10-4	1.85	Ptprg	protein tyrosine phosphatase, receptor type, G	−2.55
Ccne1	cyclin E1	1.84	Dis3l2	DIS3 mitotic control homolog (S. cerevisiae)-like 2	−2.55
Serpina3i	serine (or cysteine) peptidase inhibitor, clade A, member 3I	1.84	Cdk14	cyclin-dependent kinase 14	−2.52
Prl7d1	prolactin family 7, subfamily d, member 1	1.84	Fhod3	formin homology 2 domain containing 3	−2.50
Scn10a	sodium channel, voltage-gated, type X, alpha	1.84	Clybl	citrate lyase beta like	−2.48
S100a7a	S100 calcium binding protein A7A	1.83	Ror1	receptor tyrosine kinase-like orphan receptor 1	−2.48
Tmprss11d	transmembrane protease, serine 11d	1.83	Setbp1	SET binding protein 1	−2.47

FDR < 0.01.

**Table 5:** List of top 20 up and downregulated genes in 48 h cisplatin group.

Gene Name	Gene Description	Fold Change	Gene Name	Gene Description	Fold Change
Txnip	thioredoxin interacting protein	12.18	Ptn	pleiotrophin	−8.98
Nppb	natriuretic peptide type B	10.60	Gpc6	glypican 6	−6.49
Btg2	B cell translocation gene 2, anti-proliferative	9.11	Angpt1	angiopoietin 1	−5.96
Cdkn1a	cyclin-dependent kinase inhibitor 1A (P21)	8.43	Cpq	carboxypeptidase Q	−4.95
Atf3	activating transcription factor 3	5.83	Npr3	natriuretic peptide receptor 3	−4.58
Trp53inp1	transformation related protein 53 inducible nuclear protein 1	5.77	Slc4a4	solute carrier family 4 (anion exchanger), member 4	−4.53
S100a7a	S100 calcium binding protein A7A	5.56	Kcnip1	Kv channel-interacting protein 1	−4.48
Eda2r	ectodysplasin A2 receptor	5.54	Ror1	receptor tyrosine kinase-like orphan receptor 1	−4.32
Mdm2	transformed mouse 3T3 cell double minute 2	5.44	Car3	carbonic anhydrase 3	−4.19
Fos	FBJ osteosarcoma oncogene	5.25	Setbp1	SET binding protein 1	−4.17
Hba-a2	hemoglobin alpha, adult chain 2	5.06	Gas1	growth arrest specific 1	−4.10
Tnfsf18	tumor necrosis factor (ligand) superfamily, member 18	4.87	Sprr2g	small proline-rich protein 2G	−4.08
Ptgs2	prostaglandin-endoperoxide synthase 2	4.87	Tenm3	teneurin transmembrane protein 3	−3.96
Nr4a1	nuclear receptor subfamily 4, group A, member 1	4.61	Lphn2	latrophilin 2	−3.88
Tcrg-C4	T cell receptor gamma, constant 4	4.17	Magi1	membrane associated guanylate kinase, WW and PDZ domain containing 1	−3.85
Dynap	dynactin associated protein	4.06	Cdh18	cadherin 18	−3.83
Egr1	early growth response 1	3.97	Plxna2	plexin A2	−3.82
Snora75	small nucleolar RNA, H/ACA box 75	3.95	Atg10	autophagy related 10	−3.81
Gdf15	growth differentiation factor 15	3.88	Sprr2a2	small proline-rich protein 2A2	−3.78
Fas	Fas (TNF receptor superfamily member 6)	3.66	Ptprg	protein tyrosine phosphatase, receptor type, G	−3.72

FDR < 0.01.

**Table 6:** List of top 20 up and downregulated genes in 48 h metformin-pretreated group.

Gene Name	Gene Description	Fold Change	Gene Name	Gene Description	Fold Change
r7b	trace amine-associated receptor 7B	2.38	Csf2	colony stimulating factor 2 (granulocyte-macrophage)	−2.49
H2-DMb2	histocompatibility 2, class II, locus Mb2	2.31	Mcpt9	mast cell protease 9	−2.37
Rhox3f	reproductive homeobox 3F	2.22	Slc40a1	solute carrier family 40 (iron-regulated transporter), member 1	−2.34
Cdsn	corneodesmosin	2.21	Snora75	small nucleolar RNA, H/ACA box 75	−2.30
Scgb2b20	secretoglobin, family 2B, member 20	2.20	Tnfsf18	tumor necrosis factor (ligand) superfamily, member 18	−2.28
Ear1	eosinophil-associated, ribonuclease A family, member 1	2.15	mt-Tf	mitochondrially encoded tRNA phenylalanine	−2.16
Krt6b	keratin 6B	2.13	Cdon	cell adhesion molecule-related/down-regulated by oncogenes	−2.10
Snord98	small nucleolar RNA, C/D box 98	2.12	Tcrg-C4	T cell receptor gamma, constant 4	−2.08
Mterf1b	mitochondrial transcription termination factor 1b	2.11	Snora30	small nucleolar RNA, H/ACA box 30	−2.05
Klk1b27	kallikrein 1-related peptidase b27	2.09	Fos	FBJ osteosarcoma oncogene	−2.05
Eda	ectodysplasin-A	2.08	Btg2	B cell translocation gene 2, anti-proliferative	−2.04
Atp6v1g3	ATPase, H+ transporting, lysosomal V1 subunit G3	2.07	Amd2	S-adenosylmethionine decarboxylase 2	−2.01
Mpeg1	macrophage expressed gene 1	2.03	Mnd1	meiotic nuclear divisions 1 homolog (S. cerevisiae)	−1.99
Traj25	T cell receptor alpha joining 25	2.03	Arxes2	adipocyte-related X-chromosome expressed sequence 2	−1.99
Akr1c18	aldo-keto reductase family 1, member C18	2.02	Egr1	early growth response 1	−1.97
Ffar2	free fatty acid receptor 2	1.95	Gpx2-ps1	glutathione peroxidase 2, pseudogene 1	−1.93
Dlx6os1	distal-less homeobox 6, opposite strand 1	1.94	Arfip1	ADP-ribosylation factor interacting protein 1	−1.92
Spata16	spermatogenesis associated 16	1.91	Rhox4f	reproductive homeobox 4F	−1.90
Zfp438	zinc finger protein 438	1.90	Ampd1	adenosine monophosphate deaminase 1	−1.89
Cd27	CD27 antigen	1.89	Nr4a2	nuclear receptor subfamily 4, group A, member 2	−1.89

FDR < 0.01.

**Table 7:** List of involved genes which are expressed oppositely in 48 h cisplatin group and 48 h metformin pretreated group.

Cisplatin vs. Control	Fold Change	Metformin + Cisplatin vs. Cisplatin	Fold Change
Ampd1	2.169397	Ampd1	−1.893582
Atf3	5.831965	Atf3	−1.753464
Btg2	9.106313	Btg2	−2.038355
Csf2	3.582915	Csf2	−2.488886
Cxcl1	2.812806	Cxcl1	−1.789373
Dffb	1.934398	Dffb	−1.763047
Egr1	3.969056	Egr1	−1.966299
Fos	5.247514	Fos	−2.049006
Fosl1	2.001054	Fosl1	−1.735427
Gabra4	1.834919	Gabra4	−1.870577
Gpx2-ps1	1.643713	Gpx2-ps1	−1.931494
Hist1h2bc	1.921256	Hist1h2bc	−3.030378
Hist1h2bg	1.528113	Hist1h2bg	−2.196096
Hyal3	2.210002	Hyal3	−1.743591
Ifnz	3.560730	Ifnz	−1.791521
Ighv1-59	1.670384	Ighv1-59	−2.564053
Igkv2-116	1.526292	Igkv2-116	−1.904914
Igkv4-57-1	1.920516	Igkv4-57-1	−2.084605
Klk1b26	1.637801	Klk1b26	−1.847820
Mir335	2.549508	Mir335	−2.489257
Mir669d	3.325815	Mir669d	−2.622897
Nr4a2	2.843824	Nr4a2	−1.890484
Rhox4f	1.639365	Rhox4f	−1.898595
Slc40a1	1.579318	Slc40a1	−2.344163
Snora75	3.949902	Snora75	−2.300169
Tcrg-C4	4.166688	Tcrg-C4	−2.079953
Tnfsf18	4.874364	Tnfsf18	−2.282116
Trav6-3	1.877071	Trav6-3	−1.724728
Txnip	12.184051	Txnip	−1.879019
Vmn2r115	2.249487	Vmn2r115	−1.745398

FDR < 0.01.

## Data Availability

All the data are available in the manuscript.

## Supplementary Materials

The following supporting information can be downloaded at: https://www.mdpi.com/article/10.3390/cimb45010021/s1. Supplementary Table S1. Analysis of enrichment of KEGG pathways and involved genes in 24 h cisplatin group. Supplementary Table S2. Analysis of enrichment of KEGG pathways and involved genes in 48 h cisplatin group. Supplementary Table S3. Involved genes which are expressed oppositely in 24 h cisplatin group and 24 h metformin pretreated group.
